# The role of host defences in Covid 19 and treatments thereof

**DOI:** 10.1186/s10020-020-00216-9

**Published:** 2020-09-29

**Authors:** Maurizio Dattilo

**Affiliations:** R&D Director Parthenogen, Lugano, Switzerland

**Keywords:** Hydrogen sulfide, SARS-Cov-2, Covid 19, Heme oxygenase 1, Carbon monoxide

## Abstract

Hydrogen sulfide (H_2_S) is a natural defence against the infections from enveloped RNA viruses and is likely involved also in Covid 19. It was already shown to inhibit growth and pathogenic mechanisms of a variety of enveloped RNA viruses and it was now found that circulating H_2_S is higher in Covid 19 survivors compared to fatal cases. H_2_S release is triggered by carbon monoxide (CO) from the catabolism of heme by inducible heme oxygenase (HO-1) and heme proteins possess catalytic activity necessary for the H_2_S signalling by protein persulfidation. Subjects with a long promoter for the *HMOX1* gene, coding for HO-1, are predicted for lower efficiency of this mechanism. SARS-cov-2 exerts ability to attack the heme of hemoglobin and other heme-proteins thus hampering both release and signalling of H_2_S. Lack of H_2_S-induced persulfidation of the K_ATP_ channels of leucocytes causes adhesion and release of the inflammatory cytokines, lung infiltration and systemic endothelial damage with hyper-coagulability. These events largely explain the sex and age distribution, clinical manifestations and co-morbidities of Covid-19. The understanding of this mechanism may be of guidance in re-evaluating the ongoing therapeutic strategies, with special attention to the interaction with mechanical ventilation, paracetamol and chloroquine use, and in the individuation of genetic traits causing increased susceptibility to the disruption of these physiologic processes and to a critical Covid 19. Finally, an array of therapeutic interventions with the potential to clinically modulate the HO-1/CO/H_2_S axis is already available or under development. These include CO donors and H_2_S donors and a boost to the endogenous production of H_2_S is also possible.

## Introduction

The ongoing pandemic of Covid 19 is predicted to last several years and to produce new epidemic waves if social distancing remains the only tool to combat its diffusion (Kissler et al. 2020). Pending the availability of a vaccine, we urge to better understand the pathogenesis of the disease to develop preventive and therapeutic strategies and to individuate those subjects at higher risk for a clinically serious or fatal disease. The available epidemiologic data support the idea that the severity of the disease is lower in younger people (Verity et al. 2020) and in women (Jin et al. 2020) but no convincing explanation has been provided. The disease is more severe and/or fatal in subjects carrying other illnesses including hypertension, diabetes, cardiovascular diseases and respiratory diseases (Zheng et al. 2020). However, overt Covid 19 disease requiring hospitalization and eventually intensive care develops only in a minority of the exposed subjects with many people carrying a pauci-symptomatic or even subclinical diseases. A population screening based on nasopharyngeal swab was conducted in a small Italian village and reported that 43% of the infected population was asymptomatic although carrying a virus titre of the same magnitude of the affected patients (Lavezzo et al. 2020). The reasons why such a large part of the exposed subjects does not develop the disease is currently unknown as well as little is understood on the role of the underlying diseases, i.e. whether they simply sum-up to the Covid 19 damage or specific pathogenetic synergies occur. However, it is reasonable to postulate that the infection remains asymptomatic in subjects enjoying better robust natural defences and that these defences undergo a process of ageing as many other physiologic mechanisms. This leads our attention to the current knowledge on these physiologic defences.

### Hydrogen sulphide (H_2_S) as natural defence against viral infections

Hydrogen sulphide (H_2_S) is the third discovered gasotransmitter, besides nitric oxide (NO) and carbon monoxide (CO). The 3 gasotransmitters interact each other (either synergy or antagonism) in a district and time specific manner that escapes our full understanding. They regulate basic homeostatic functions including oxy-redox balance, bioenergetics, endothelial function/vasodilation, coagulation and platelets functions and others (Giuffre and Vicente 2018).

H_2_S has been described as a main human natural defence against infections of the airways from encapsulated RNA viruses. A first study on the model of Respiratory Sincitial Virus (RSV) and other paramyxovirus infections in airway epithelial cells showed that the early phase of the infection associates with a fall of production and of intracellular levels of H_2_S (Li et al. 2015). The inhibition of a H_2_S producing enzyme, Cystathionine Gamma Lyase (CSE), further promoted viral invasion whereas the application of the H_2_S donor GYY4137 significantly reduced proinflammatory mediator production and viral replication by inhibiting syncytium formation and virus assembly/release. These results were confirmed in vivo in a mice model of RSV infection using intranasal administration of GYY4137 (Ivanciuc et al. 2016). The H_2_S donor GYY4137 was then shown, in in-vitro cell models, to inhibit the inflammatory mechanisms and the replication of an array of enveloped RNA viruses from Ortho-, Filo-, Flavi- and Bunyavirus families including all the influenza virus strains tested (H1N1, H3N2 and Brisbane strain) (Bazhanov et al. 2017). However, in the study from Li et al. (2015) GYY4137 had been used in millimolar concentration, which is quite high. The same authors therefore tested the activity of another H_2_S donor, Thiol-Activated gem-Dithiol-based H_2_S donor (TAGDD), that releases H_2_S only in biological fluids, and achieved dose-dependent activity against RSV in-vitro at micromolar concentration (range 10–100 μM) and in-vivo in mice at the dose of 1 mg/kg body weight of TAGDD (Bazhanov et al. 2018). Accordingly, H_2_S modulation was proposed as an approach for broad spectrum antiviral treatments. SARS-CoV-2 is as well an encapsulated RNA virus and might respond to the same mechanisms. Indeed, in Covid 19 patients the serum concentration of H_2_S is higher in survivors compared to fatal cases and inversely correlates with the level of IL-6 and with mortality (Renieris et al. 2020).

### H_2_S release and signalling

H_2_S is produced by three different enzymes: Cystathionine Beta Synthase (CBS) and Cystathionine Gamma Lyase (CSE), both requiring vitamin B6 as necessary co-factor, and 3-Mercaptopyruvate Sulfur Transferase (3MST) that is not dependant on B6. All of these enzymes belong to the transsfulfuration pathway that is responsible for the metabolism of sulfur amino acids and that plays a key role in the endogenous antioxidant system. According to the canonical pathway, the first enzyme, CBS, binds serine to homocysteine, resulting from methionine de-methylation, and forms cystathionine. This is cleaved by CSE into α-ketobutyrate, ammonia and cysteine, which can be used for protein synthesis and for the synthesis of glutathione (GSH) to feed the endogenous antioxidant system. No H_2_S is released in these reactions. Cysteines can be also transaminated to 3-Mercaptopyruvate that is used by 3MST to generate H_2_S. However, 3MST produces H_2_S only in mitochondria whereas the majority of H_2_S is produced by CBS and CSE when working according to their alternative pathways instead of the canonical ones (Kabil and Banerjee 2014). Indeed, CBS and CSE exert unspecific recognition of their substrates and are able to function is a sort of reverse manner, which was called alternative pathways of CBS and CSE (Singh et al. 2009): CBS mainly produces H_2_S from cysteine forming lanthionine; CSE mainly produces H_2_S from homocysteine forming homolanthionine.

**Fig. 1:**
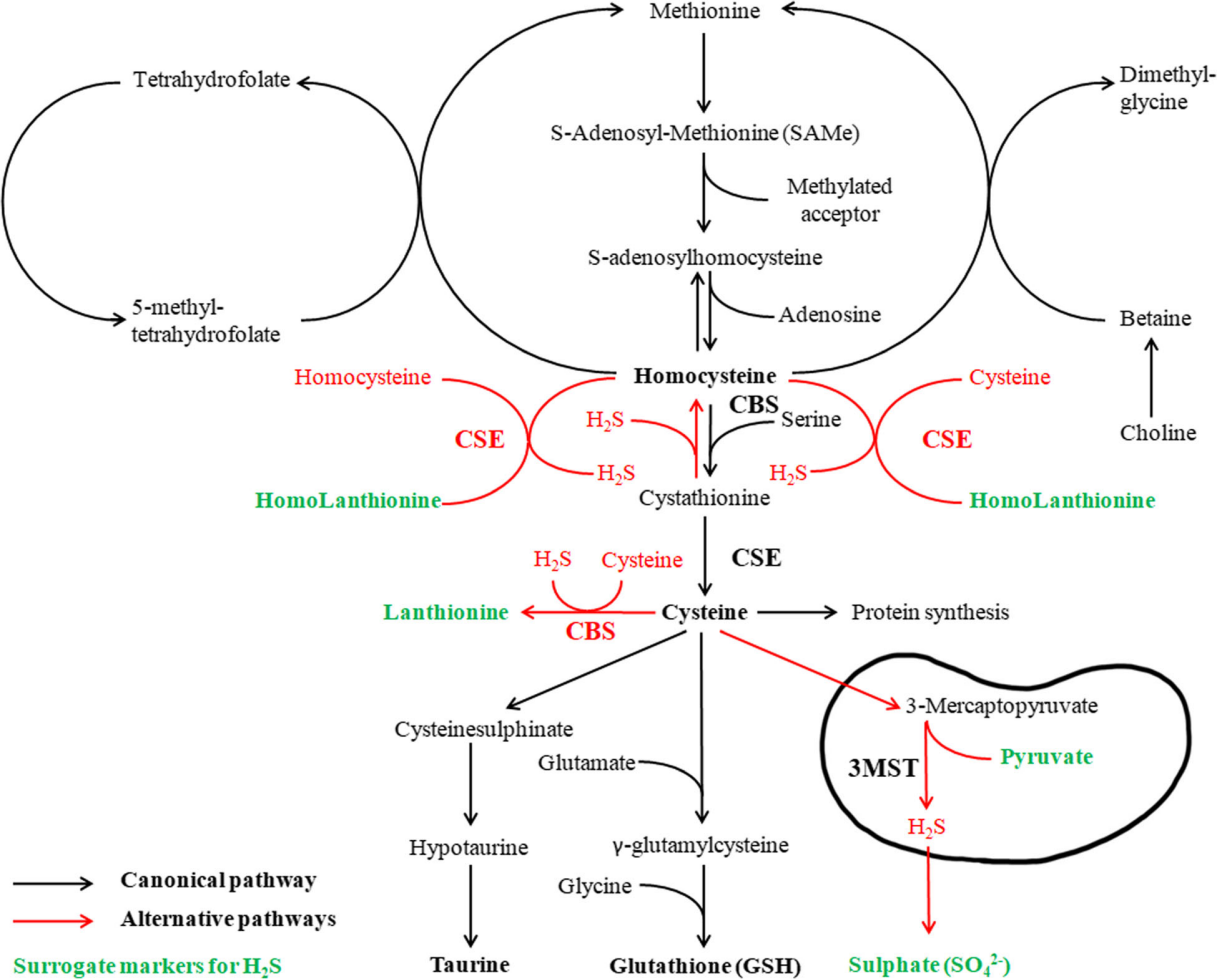
Canonical and alternative transsulfuration pathways. The canonical pathway, i.e. transulfuration of homocysteine to cysteine and then GSH synthesis, are shown by black lines; alternative pathways, leading to H_2_S release, are shown by red lines. The products of H_2_S releasing reactions, functioning as surrogate markers of H_2_S release are shown in green colour: Lanthionine reports on H_2_S from CBS reactions; Homolanthionine reports on H_2_S from CSE reactions; Pyruvate reports on intramitochondrial release by 3MST; Sulphates (SO42-) reports on H_2_S oxidation on the respiratory chain

H_2_S can be detected from biological samples by a variety of methods based on fluorescent probes (Lin et al. 2015), which can be coupled with microfluidic systems to reduce the biases from the time dependence of the detected reactions (Karunya et al. 2019). These methods are reliable tools to monitor H_2_S in vitro but their clinical use may have limited value. H_2_S has an extremely short half-life (seconds to minutes) (Polhemus and Lefer 2014) and acts in a paracrine manner so that the sphere of action of sulfide produced by a single cell expands to involve more than 200 neighbouring cells (Cuevasanta et al. 2012). Accordingly, the amount of H_2_S detected, e.g. in plasma, at a given time point may have very little relation to the actual previous function of the related pathways, e.g. in the cardiovascular system, over an extended period of time. For clinical monitoring purposes we consider a lot more informative the detection of the molecules produced by the H_2_S-releasing reactions that are stable over time, accumulate in circulation and can be measured by Liquid Chromatography Mass Spectrometry (LC-MS/MS) (Kozich et al. 2016; Perna et al. 2017). See Fig. 1 for a schematic representation of canonical and alternative transsulfuration pathways and surrogate markers of H_2_S production.

The mechanisms causing CBS and CSE to shift from canonical to alternative reactions are not fully understood but are likely related to the interaction of these enzymes with the other two gasotransmitters, NO and CO. Both NO and CO have the capacity to bind the CBS heme group, which results in an inhibition of the canonical CBS activity, whereas increasing oxygen concentration oxidizes the heme and reactivates the enzyme. The inhibition of CBS by NO and CO will result in increased H_2_S release from CBS and even more from CSE as the result of changed concentration of CSE substrates (Banerjee 2017). In particular, it has been shown that the switch from canonical to alternative reactions occurs as a response to Endoplasmic Reticulum (ER) stress and is mediated by the activation of the inducible heme oxigenase (HO-1) and the consequent release of CO (Kabil et al. 2016). CO released by HO-1 binds CBS and the resulting inhibition causes decreased production of GSH while the SH groups are preferentially converted to H_2_S by CSE reactions. Based on these data, HO-1 is a main activator of H_2_S generation.

The standard two electron redox potential of H_2_S/S0 couple, at pH 7, versus the standard hydrogen electrode, is − 0.280 V (Mishanina et al. 2015) and is not far from that of glutathione disulfide/glutathione (E°′ = − 0.262 V), thus it is a reducing substance. However, stochiometric considerations do not support a primary role of H_2_S as a free radical scavenger (Xie et al. 2016a). Rather, H_2_S is supposed to exert its biological effects by modification of structural and functional proteins. First, H_2_S reacts with the metal centres of a variety of heme-proteins including globins, cytochrome c oxidase, catalase, peroxidases and CBS by reducing the heme iron. Second, H_2_S reacts with protein cysteines to form persulfides, i.e. blockade of the protein cysteine in its persulfide form (protein-S-SH) (Giuffre and Vicente 2018), which changes the function of the protein. Persulfidation signalling is known to be responsible for primary activities of H_2_S. The persulfidation of cysteines of the Nuclear Transcription Factor Y Subunit Beta (NFYB) by H_2_S is responsible for the activation of the ten-eleven translocation enzymes Tet1 and Tet2 to mediate Foxp3 demethylation downstream of TGF-b and IL-2 signalling, thus promoting T regulatory (Treg) cell function and immune homeostasis (Yang et al. 2015). In practice, H_2_S helps converting innate immunity toward balanced reactions, including antibody responses. Moreover, persulfidation of the K_ATP_ channels is involved in the regulation endothelial function. H_2_S released by both endothelial and smooth muscle cells diffuses into the small vessels and acts on leucocytes to keep open the K_ATP_ channels (Zanardo et al. 2006). In this condition, leucocytes are not able to adhere to the endothelium and to trigger the inflammatory cascade. Opposite, any drop of H_2_S is followed by leucocyte adhesion and inflammation.

Circulating globins have a primary role in the transduction of H_2_S into persulfidation signals by exerting a clinically relevant enzymatic activity responsible for the non-canonical oxidation of H_2_S to produce thiosulfates (SSO_3_), the actual final effector of protein persulfidation. Bilska-Wilkosz et al. (2017) demonstrated that most of the conversion of H_2_S to thiosulfate is generated by the enzymatic activity of haemoglobin, myoglobin and neuroglobin. Thus, heme globins are at the same time targets and effectors of H_2_S and any derangements of their concentration, structure and function is predicted to deeply affect the H_2_S system.

### Heme oxygenase 1 (HO-1), carbon monoxide (CO) and heme metabolism

Human heme catabolism is accomplished by the inducible heme oxigenase (HO-1) and, likely to a lesser extent, by the constitutional HO-2 (Duvigneau et al. 2019). HO-1 metabolizes the heme group of a variety of heme-proteins including myoglobin, neuroglobin, cytochrome c, cytochrome p450, nitric oxide synthases, and guanylate cyclase. The catabolism of the heme by HO-1 produces biliverdin, ferrous iron, and CO. Once released by HO-1 activity, CO is able to bind other molecules of hemoglobin forming carboxyhemoglobin that has a lower affinity for oxygen so that the release of CO is followed by a relative increase of tissue oxygen release to counteract local hypoxia.

Steady state expression of the *HMOX1* gene, coding for HO-1, is quite low. The gene can be activated by a very wide variety of stimuli, all of them in some ways carrying oxidative stress, including hyperoxia, hypoxia, heat shock, endotoxin, hydrogen peroxide, cytokines, UV light, heavy metals, and nitric oxide. Not surprisingly, being the organ for the exchange of gaseous molecules with heme proteins, the lung is a primary site of expression and activity of HO-1 where it exerts protective actions (Morse and Choi 2002). Interestingly, decreased HO-1 activity has been directly related to the susceptibility to many viral infections, including Zika virus (El Kalamouni et al. 2019), influenza virus (Cummins et al. 2012), Dengue (Tseng et al. 2016), human immunodeficiency virus (HIV) (Gill et al. 2018), and hepatitis B (Protzer et al. 2007).

### SARS-CoV-2, heme metabolism and Covid 19

A preliminary, unpublished report from Liu & Li (2020) used a computer assisted simulation to predict the biological roles of specific viral proteins of SARS-CoV-2. They found that the virus can bind to heme porphyrins by its ORF8 and surface glycoprotein and inhibit human heme metabolism. According to the Authors, this may cause shortage of oxygen and carbon dioxide-transporting hemoglobin predisposing to respiratory distress. The proposed model was entirely speculative and caused several criticisms from the readers leading the Authors to release a new version of the manuscript leading to a partial retraction of the initial statements, although confirming the main interpretation (Liu and Li 2020v1). In this new form the report of Liu and Li (2020v8) remained questionable due to the complete absence of experimental evidences, to possible methodological issues and to lack of an interpretative model fitting with the current hypotheses (Read 2020).

**Fig. 2:**
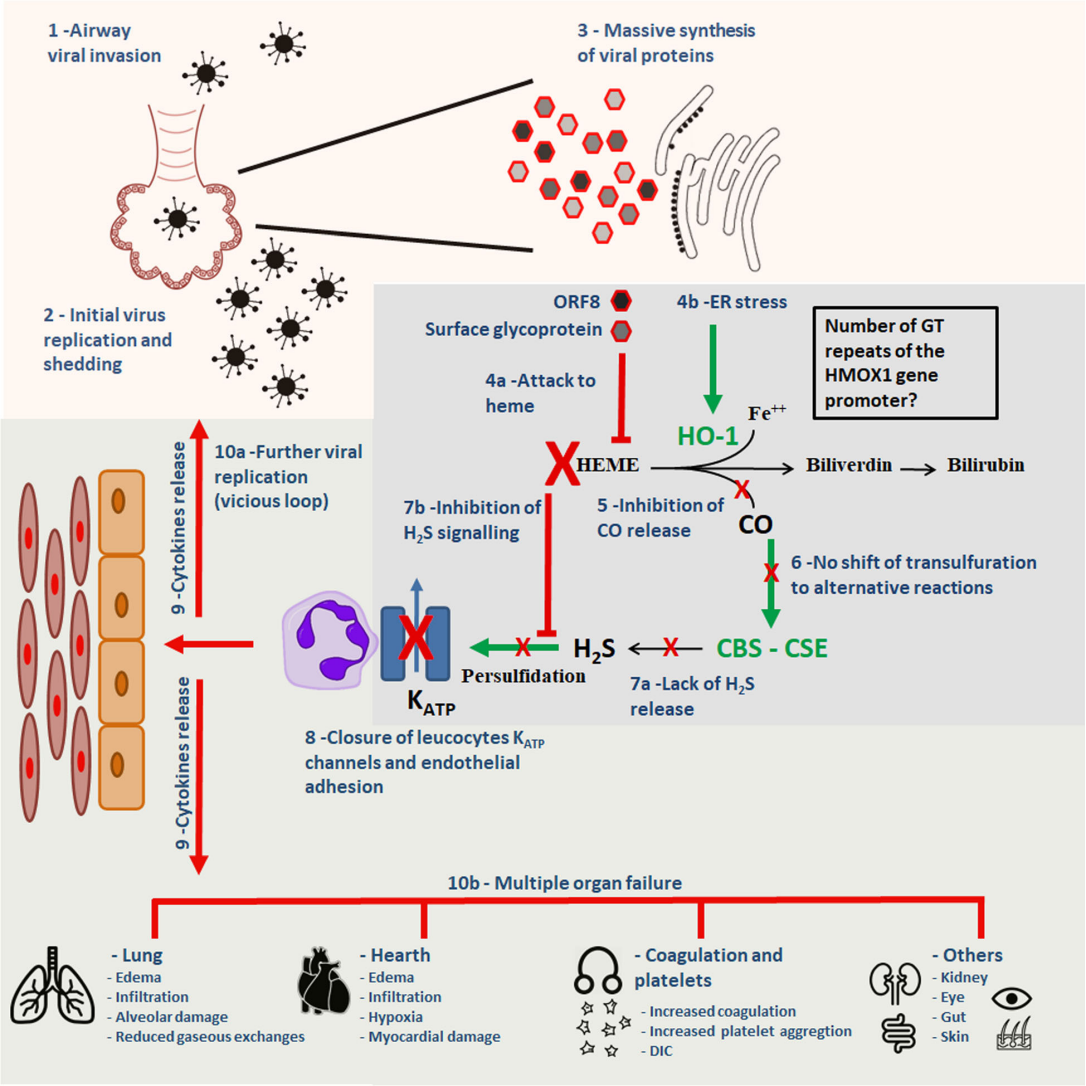
Proposed model for the pathogenesis of Covid 19. 1 - The virus penetrates the airways and infects the bronchial and alveolar epithelium, other routes of invasion are possible (e.g. eye); 2 - The virus starts replication into the cell; 3 - Massive synthesis of viral proteints into the endoplasmic reticulum (ER); 4 - The ORF8 and surface glycoprotein start attacking and destroying the heme of heme proteins (4a) while the ER stress caused by massive protein synthesis activates the physiologic HO-1 response (4b); 5 - The activation of HO-1 fails to work due to substrate (heme) inhibition by viral proteins; 6 - In absence of CO from HO-1 there is no shift of transulfurations to alternative reactions and failure to release H_2_S; 7 – Hampered H_2_S release from CBS/CSE (7a) inhibits their persulfide forming activity while destruction of the heme by viral proteins (7b) further decreases H_2_S effects by inhibiting its persulfidation signalling; 8 - Due to low H_2_S-induced persulfidation, the K_ATP_ channels of leucocytes closes down, which triggers leucocyte adhesion to the endothelium; 9 - The adhesion of leucocytes is followed by massive release of inflammatory cytokines, both local and systemic; 10a - Inflammation induced by cytokines further feeds viral replication amplifying the effects in a vicious loop; 10b - Inflammatory cytokines diffuse systemically and cause or exacerbate pre-existing damges in many organs and functions, mainly hearth and coagulation. Black box: Subjects with a strong HO-1 reaction (4b) are predicted to overcome the viral aggression and to exert an asymptomatic or pauci-symptomatic disease; Subjects with a defective genetic background, e.g. weak expression of the *HMOX1* gene coding for HO-1, may suffer critical or fatal Covid 19

However, the disruption of heme metabolism may have profound effects on the metabolism of gasotransmitters leading to a series of consequences that may resemble the actual clinical and pathologic findings from Covid 19. The destruction of heme porphyrins as hypothesized by Liu & Li (2020v1; 2020v8) is able to deeply perturb the HO-1/CO/H_2_S axis leading to the disruption of the host defences against SARS-CoV-2: First, this mechanism would decrease the availability of the heme substrate for the HO-1 activity with reduced release of CO; Second, the lack of CO signalling will reflect in no activation of the CBS and CSE alternative reactions for the release of H_2_S as described by Kabil et al. (2016); Third, the destruction of heme globins will also hamper the main system for transduction of H_2_S in persulfidation signalling (Bilska-Wilkosz et al. 2017), resulting in a further depression of H_2_S effects. Moreover, and independently of any interference with H_2_S release, the lack of CO within affected tissues will not allow adaptation to hypoxia with further shortage of tissue oxygenation. This model is of particular relevance to SARS-CoV-2 infection because viral infections are known to generate ER stress as an invasion mechanism (Choi and Song 2020), likely by overloading the protein synthesis machinery with the production of capsid proteins. We postulate that SARS-CoV-2 infection generates ER stress just like other viruses, but the direct antagonism to HO-1 activity does not allow the reaction by CO/H_2_S release with deleterious effects in subjects with constitutional low reactivity of the HO-1/CO/H_2_S axis. The proposed model for the pathogenesis of Covid 19 from SARS-CoV-2 is depicted in Fig. 2.

Thus, subjects infected by SARS-CoV-2 that are able to respond to the viral aggression with a robust reaction of the HO-1/CO/H_2_S axis, i.e. to keep open the K_ATP_ channels of the leucocytes circulating in the lung and elsewhere as described by Zanardo et al. (2006), would prevent the trigger to the inflammatory cascade necessary for viral replication. Opposite, subjects with a weak reaction of the same axis to SARS-CoV-2 infection are expected to undergo a generalized endothelial dysfunction with interstitial lung infiltration and systemic derangement of the coagulation and platelet function, which resembles the pathologic picture of aggressive Covid 19 (Salerno et al. 2020).

### Role of the HO-1/CO/H_2_S axis in Covid 19

In summary, the SARS-CoV-2 responsible for Covid 19 may have as the primary invasive mechanism the disruption of heme proteins. Based on a wide array of evidences from the scientific literature, this is predicted to result in blockade of the natural defences of the airways, activation of the inflammatory cascade by leucocyte adhesion, cytokines release and interstitial infiltration hampering gaseous exchanges. Pending the demonstration of this model by dedicated, prospective studies, the proposed model might actually fit with most of the clinical features of Covid 19, of its epidemiology and co-morbidities.

#### Age and sex ratio of Covid 19

The median age of hospitalized patients was reported around 47 years with a male sex rate of 52% in the Far East (Guan et al. 2020) and increased to 63 years with 82% male rate in Europe (Grasselli et al. 2020). However, independently of the mean age of presentation, it is evident that the fatality rate of Covid 19 shows a strong age gradient with increased lethality with increasing age (Verity et al. 2020). The incidence of the disease appears similar between sexes, however men with Covid-19 are more at risk for worse outcomes and death compared to women, independent of age (Jin et al. 2020). There is no fixed explanation for the age and sex distribution of Covid 19 but it has been observed that older age and male sex are independently related to changes in the performance of the immune system with a decline in adaptive immunity, mainly of Treg lymphocytes, paralleled by an activation of innate immunity and this resembles what is seen in Covid 19 (Márquez et al. 2020).

On the train of the above observations, we wish to underscore that H_2_S is both an anti-aging mechanism and a hallmark of aging (Perridon et al. 2016) and the progressive decline of its availability may explain the age pattern of Covid 19. Indeed, H_2_S is to our knowledge the best characterized inducer of Treg lymphocyte differentiation (Yang et al. 2015) and any decline of its release associates with derangement of innate immunity. The ability to release H_2_S is also increased in women due to a direct effect of estrogens on the activity of hepatic and endothelial CSE and this was proposed as a main reason for the lower burden of cardiovascular disease in fertile women (Li et al. 2017). Accordingly, pre-menopausal women, although being infected by SARS-CoV-2 at the same rate as men, may have better effective natural reactions leading to a less severe clinical impact. In summary, the known patterns of age and sex related H_2_S production may fit with the age and sex distribution of Covid 19.

#### Clinical manifestations of Covid 19

Covid 19 is an airway-acquired viral systemic disease with multi-organ failure and a variety of co-morbidities (Renu et al. 2020). The most common presenting symptoms are cough, fever, dyspnea, myalgias, diarrhea, nausea and vomiting whereas the laboratory findings report lymphopenia, thrombocytopenia, elevated liver-function values and inflammatory markers (Goyal et al. 2020). The level of respiratory distress, up to the development of an Acute Respiratory Distress Syndrome (ARDS) eventually requiring mechanic ventilation, has been directly related to the release of inflammatory cytokines, mainly IL-6 (Gubernatorova et al. 2020). The administration of H_2_S in an animal model of acute lung injury has been shown to decrease IL-6 secretion in parallel with reduction of lung oedema and respiratory impairment (Li et al. 2008). H_2_S is indeed a direct inhibitor of IL-6 release and this effect is related to the action on K_ATP_ channels (Fouad et al. 2020). Accordingly, a study investigating the serum H_2_S concentration in Covid 19 patients found that survivors had significantly higher H_2_S levels on day 1 and 7 after admission compared to fatal cases and that H_2_S inversely correlated with the level of IL-6 and with mortality (Renieris et al. 2020). Therefore, there may be a relation between the level of impairment of H_2_S release and the severity of Covid 19 respiratory symptoms, which should be duly investigated.

#### Olfactory and gustative symptoms and conjunctivitis in Covid 19

Patients with Covid 19 report perturbations of taste and smell sense. According to Mao et al. (2020) this occurred in 5–6% of hospitalized patients. Thereafter, Giacomelli et al. (2020) reported these symptoms in 20 patients out of 59 (34%) and in 6 cases (10%) they were the only symptoms. Females and young people were more likely to report such sensory impairments. Worth to note the concentration of H_2_S in saliva is in the low micromolar range (1–7 μMol), i.e. the highest known (Zaorska et al. 2018). It serves to activate salivary carbonic anhydrase (Bhakta et al. 2016) and in turn to regulate the salivary-gastric recirculation of nitrites/NO (Aamand et al. 2009). Salivary Carbonic Anhydrase VI (CA-VI) is also known as “gustin”, i.e. it is found on the tongue papillae where it serves as receptor for bitter taste (Patrikainen et al. 2014). Patients with loss and/or distortion of taste and smell after influenza-like symptoms were shown to have decreased amounts of gustin/CA-VI and histologic changes in their gustative papillae (Henkin et al. 1999). Thus, modified perception of taste is expected to occur at time of viral invasion of the airway by SARS-CoV-2 as consequence of the fall of H_2_S as well as in the healing phase as consequence of a rebound increase of H_2_S. The same rebound increase of H_2_S in the recovery phase is likely responsible for the smell disturbancies of Covid 19. Indeed, higher amounts of H_2_S have been linked to the so-called olphactory fatigue that can evolve up to loss of smell (Hirsch and Zavala 1999).

SARS-CoV-2 infection involves the eye both as entry point and as site of viral shedding (Zhong et al. 2020). Conjunctivitis is frequently reported in Covid 19 patients and can be the only symptom in clinically mild cases (Scalinci and Battagliola 2020). There is no information on the concentration of H_2_S in lacrimal secretion, however it is expected to be present and to exert the same barrier role against viral infections. Already three centuries ago H_2_S was described by the Italian physician Bernardino Ramazzini as irritating for the eye and able to cause conjunctivitis in case of exposure (Szabo 2018). Accordingly, conjunctivitis in Covid 19 may be consequence of direct viral damage and anticipate a serious disease. However, it may be worth to investigate if, when presenting as an isolated symptom or during the evolution of milder cases, eye irritation can be a sign of rebound hypersecretion of H_2_S marking the recovery phase.

In summary, the olfactory and taste perturbations, as well as isolated conjunctival problems, reported by Covid 19 patients might be linked to a hyperactivation of the H_2_S system that characterizes cases with strong and positive reaction to the infection and may result of guidance in the clinical management, i.e. to individuate cases predicted for milder evolution at time of triage.

#### Co-morbidities of Covid 19

Besides older age, risk factors for severe or fatal Covid 19 include d-dimer greater than 1 μg/mL and higher Sequential Organ Failure Assessment (SOFA) score on admission (Zhou et al. 2020). Patients with a higher SOFA may be at increased risk just because of unspecific contribution of underlying diseases to the general health status, however most of the described co-morbidities are known to be related to a failure of H_2_S release and it is possible that severely affected patients carry predisposing factors, e.g. genetics, that are both among the causes for their pre-existing illnesses and for a more severe progression of Covid 19.

Cardiovascular disease (CVD) is associated with an increased risk of in-hospital death among patients hospitalized with Covid-19 (Cheng et al. 2020). According to the guidance document of the European Society of Cardiology (The European Society for Cardiology 2020), CVD in Covid 19 may be secondary to acute lung injury, which leads to increased cardiac workload, but may also result from direct cardiac damage following cytokine release storm, mainly IL-6 and IL-17, caused by lung infection. Post-mortem analysis of Covid 19 patients revealed multi-organ endotheliitis (mainly in lung, heart, kidney, and liver) with direct viral infection of endothelial cells (Varga et al. 2020). These pathologic findings can be directly related to a primary failure of the HO-1/CO/H_2_S homeostasis. The protective role of HO-1 and its products in the heart and cardiovascular system is unequivocal (Otterbein et al. 2016). It is involved in the defences against myocardial ischemia-reperfusion injury and in vascular dysfunction, which are more common and severe in carriers of HO-1 deficient genetic variants. H_2_S, which is produced in response to HO-1/CO, has been proposed as the link between inflammation and endothelial dysfunction in CVD (Sun et al. 2020). It is produced in both the smooth muscle and endothelial cells, mainly by CSE activity, and is directly involved in the vasodilation process within the CV system in concert with NO (Wu et al. 2018). Lack of H_2_S signalling is predicted to perturbate cardiovascular homeostasis and to generate endothelial dysfunction, inflammation, deranged response to hypoxia and heart failure (Pan et al. 2017). This was confirmed in a murine model of Coxsackie virus B3 (CVB3)-induced murine myocarditis where the administration of a H_2_S donor prevented myocardial injury by up-regulating HO-1 expression and the release of CO as measured by increased carboxyhemoglobin (Zhang et al. 2017).

Covid 19 patients are also at higher risk for thrombotic disease states including acute coronary syndrome (ACS), venous thromboembolism (VTE) such as deep vein thrombosis (DVT) or pulmonary embolism (PE), and stroke (Watson et al. 1995), which fits with the prognostic value of d-dimer elevation (Zhou et al. 2020). Moreover, the clinical conditions are often complicated by the occurrence of Disseminated Intravascular Coagulation (DIC) (Klok et al. 2020; Lodigiani et al. 2020). Thromboembolic phenomena and DIC may be the consequence of H_2_S suppression. Indeed, H_2_S is a direct inhibitor of platelet aggregation (Li et al. 2016) as well as of the adhesive activity of fibrinogen and collagen (Morel et al. 2014). Interestingly, H_2_S has been shown to protect against Tissue Factor-induced Disseminated Intravascular Coagulation (DIC) in rabbits by inhibiting coagulation and platelet aggregation (Lu et al. 2015).

Kawasaki disease is a paediatric vasculitis-inflammation in blood vessel walls leading to coronary artery lesions that has been observed in severe form in kids infected by SARS-CoV-2 (Xu et al. 2020). It has been demonstrated that, in children with Kawasaki disease in acute phase, plasma H_2_S is low and its concentration inversely correlates with the occurrence of coronary artery lesions (Sun et al. 2015). In contrast, the amount of H_2_S in lymphocytes of children with Kawasaki disease was reported to be increased and to directly correlate with the occurrence of coronary artery lesions in the convalescence period (Lin et al. 2020). Very likely H_2_S had been detected in early damage phase in the first study and during the rebound hyper-secretion of H_2_S in the second study. However, it is very likely that perturbations of H_2_S metabolism following to SARS-CoV-2 infection may contribute to the observed association of Covid 19 with Kawasaki disease.

Obesity has been identified as a risk factor for symptomatic, severe and critical Covid 19 independently of other risk factors (Caussy et al. 2020). Obesity often overlaps with type 2 diabetes mellitus, which is as well associated with doubled chances of severe and fatal Covid 19 (Scheen et al. 2020). Micro and macro vascular damage in diabetes is a possible cause of susceptibility whereas the mechanic impairment of respiratory function is an obvious link between obesity and Covid 19. However, a direct role for impaired H_2_S signalling may also be in place. Animal models show that HO-1 levels and HO activity are low (Drummond et al. 2019) and that CBS, CSE and H_2_S are as well reduced in obesity (Katsouda et al. 2018). H_2_S depletion may be responsible for the loss of anti-contractile function of perivascular adipose tissue in obesity (Candela et al. 2017) and this is predicted to directly increase the clinical burden of Covid 19. Insulin was shown to decrease the expression of a main H_2_S releasing enzyme, CBS, both in vitro and in vivo (Ratnam et al. 2002) and clinical hyperinsulinemia associates to lower H_2_S in blood and to vascular inflammation (Jain et al. 2010). Accordingly, H_2_S administration to diabetic animals slows down the progression of atherosclerosis (Xie et al. 2016b) confirming a direct link between H_2_S and endothelial damage in diabetes. In summary, based on the common impairment of the HO-1/CO/H_2_S axis, the role of obesity and diabetes, independently or in association, in Covid 19 could not be a simple co-morbidity and synergistic pathogenic mechanisms seems to be in place.

### H_2_S and response of Covid 19 to current treatments

Once understood the role of a hampered HO-1/CO/H_2_S axis in the pathogenesis of Covid 19, it is worth to look at the interaction between the proposed model and the current practices in the treatment of the disease.

Recurrence to mechanical ventilation (MV) is common in case of critical Covid 19 patients developing respiratory distress with low oxygen saturation. However, the mortality rate of patients elected to MV is quite high. A large case series describing the clinical outcome of hospitalized patients in New York City reported a mortality rate for those who received mechanical ventilation of 76.4% among patients aged 18-to-65, which raised to 97.2% in those older than 65, compared to 1.98 and 26.6%, respectively, in the same age groups without MV (Richardson et al. 2020). Thus, mortality associated to MV in Covid 19 is extremely high and this may depend on already critical conditions of the patients at time of election for MV. However, a direct, negative effect of MV should be considered. Ventilation Associated Lung Injury (VILI) is a well-known and dangerous syndrome associated to MV that, although mitigated and prevented by modern adjustment of MV parameters, remains a clinical issue (Marini 2019). VILI is supposed to be induced by mechanical stresses, however it results in cytokine release and inflammation (Liu et al. 2014). A bulk of evidences from animal models indicate that H_2_S is effective in relieving damages from VILI. In particular, it has been shown that H_2_S relieves VILI by regulating autophagy and ER stress (Ge et al. 2019), which points to an involvement of the HO-1/CO/H_2_S axis in the pathogenesis of VILI. Assumed the proposed direct negative effect of SARS-CoV-2 on the same axis, Covid 19 patients may suffer a synergistic negative effect leading to an increased susceptibility to VILI once addressed to MV and this may have a role in the high mortality rates reported.

Due to side effects and to increased risk of severe bacterial complications and VTE, the use of paracetamol (acetaminophen) at recommended doses is assumed to be a safer option compared to NSAIDs for the control of the symptoms in Covid 19 patients (Micallef et al. 2020). Indeed, although paracetamol is the most common drug causing DIC (Bonaldo et al. 2017), toxicity is expected for daily doses higher than 6 g, which is easily avoided. However, it must be considered that the mechanism of paracetamol toxicity involves an interference with H_2_S activities (Wilinski et al. 2011) so that the drug and the virus might synergize resulting in a decreased toxic threshold for paracetamol. Noteworthy, in experimental models H_2_S is effective in the treatment of paracetamol-induced toxicity (Ozatik et al. 2019) and protects against Tissue Factor-induced DIC by inhibiting the activity of coagulation system and platelet aggregation (Lu et al. 2015). Based on the above, the dose safety interval and the indications for use of paracetamol in Covid 19 patients should be further evaluated. Finally, a possible contribution of this mechanism in the high rate of DIC in Covid 19 should be investigated.

Chloroquine and hydroxychloroquine are used for the treatment of Covid 19 and are undergoing clinical trials based on antiviral activity in experimental models, which was however questionable (Guastalegname and Vallone 2020). These substances are well-known and powerful inhibitors of autophagy, they hamper the autophagosome fusion with lysosomes (Mauthe et al. 2018) and this was proposed among the possible reasons for their proarrhythmic effects (van Bavel et al. 2018). Autophagy is induced by ER stress and its inhibition may be detrimental for the defences against viral infections (Rashid et al. 2015), thus inhibition of autophagy by chloroquine may have negative consequences in Covid 19. Opposite, H_2_S at physiologic amounts is a modulator of autophagy and for this reason it has been proposed as an antiarrhythmic treatment (Zhong 2010). Accordingly, the administration of chloroquine during SARS-Cov-2 infection, i.e. when autophagy should work but the activation of H_2_S is hampered, may contribute to its pro-arrhythmic effect, especially if used at high doses in subjects with pre-existing cardiovascular diseases.

### Genetic susceptibility to Covid 19

The most obvious candidate for a genetic predisposition to Covid 19 in severe form is the *HMOX1* gene coding for the HO-1 enzyme. A polymorphism in the number of GT repeats of the *HMOX1* gene promoter is known to modulate the gene expression. A small number of GT repeats in the promoter associates to intensive gene expression measured at mRNA level, the opposite for promoters with abundance of GT repeats (Walther et al. 2012).

The GT polymorphism of the *HMOX1* gene is directly linked to the susceptibility to coagulation problems and cardiovascular disease. A large case series of subjects reporting a first episode of VTE found that patients with a long (GT repeats) promoter variant of the *HMOX1* gene, vouching for less efficient activation of the HO-1/CO/H_2_S axis, had double chances to suffer a VTE recurrence (Mustafa et al. 2008). Children affected by sickle cell disease suffer vaso-occlusive episodes that can result in acute chest syndrome (ACS), pain, and stroke. Children with a short promoter had decreased risk of adverse outcomes from ACS and lower rates of hospitalization for ACS independently of their fetal hemoglobin level (Bean et al. 2012). Fewer GT repeats in *HMOX1* promoter are also associated with a lower severity score in coronary artery disease (Liang et al. 2013). A comprehensive meta-analysis concluded that the short GT genotype of the *HMOX1* promoter was associated with decreased risk of coronary artery disease after controlling for biases due to age and sex matching, extent of coronary stenosis and ethnicity (Qiao et al. 2014).

The role of HO-1 expression and activity in the susceptibility to a variety of viral infections is well known and the polymorphisms in the GT repeats of the promoter of *HMOX1* gene is a main variable (Espinoza et al. 2017). In HIV infection a longer GT segment associates to a higher amount of CD14+ monocytes and to a higher virus titre during and after antiretroviral therapy (Seu et al. 2012) and to increased neuroimmune activation and risk for encephalitis (Gill et al. 2018).

Other genes whose defective variants might cause a low efficiency of the HO-1/CO/H_2_S axis include the H_2_S-generating enzymes CBS, CSE and 3MST. Indeed, any genetic variant that modifies the expression level, the enzymatic properties and the response to the regulatory signals of these enzymes might be responsible for a lower response independently of any *HMOX1* polymorphisms, but there are no data in support and interactions are difficult to predict.

In summary, carriers of a long promoter of the *HMOX1* gene due to a larger number of GT repeats are weaker inducer of the HO-1/CO/H_2_S axis and are predicted to have increased susceptibility to Covid 19, which could be checked aiming to individuate those subjects necessitating tailored preventive measures and tailored treatments in case of infection. It must be recalled that *HMOX1* messengers can undergo post-translational and structural modifications which modulate HO-1 function (Dunn et al. 2014) and different functional phenotypes may develop with the same number of GT repeats in the promoter. Moreover, other genes have the potential to exert a main role independently of *HMOX1*. Nevertheless, the genetic information on the *HMOX1* gene promoter could be complemented with the analysis of the actual phenotype (e.g. HO-1 protein level) and concentration of the H_2_S release biomarkers (lanthionine and homolanthionine), therefore the individuation of subjects predisposed to SARS-CoV-2 damages may be possible.

### Supporting the HO-1/CO/H_2_S axis in Covid 19

**Fig. 3:**
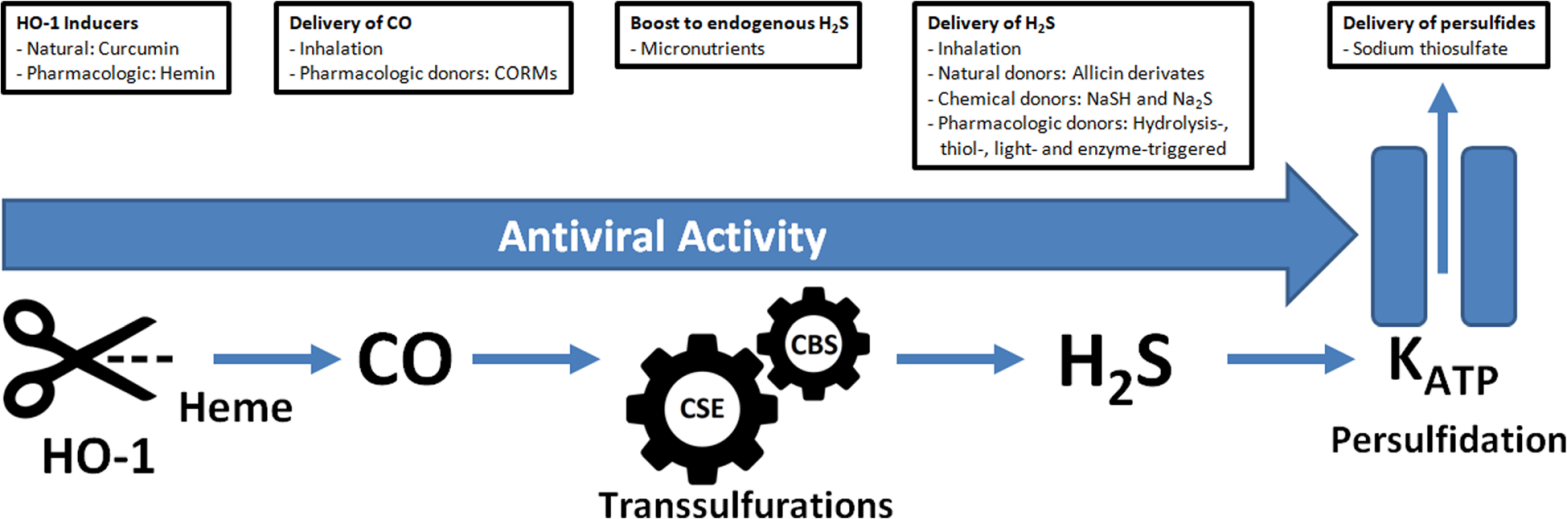
Possible interventions to boost the HO-1/CO/H2S axis. The HO-1/CO/H_2_S axis can be modulated at various steps. HO-1 inducers may increase the expression and activity of the enzyme resulting in faster activation of CO release; CO delivery may compensate a defective delivery from HO-1 reactions; A boost to the endogenous H_2_S synthesis would compensate a defective activation of the alternative pathways of CBS and CSE; Direct delivery of H_2_S may increase availability and actions from H_2_S independently of the above passages; Delivery of persulfides, although not substituting all the functions of H_2_S, may be sufficient for the action on the K_ATP_ channels. Multiple interventions targeting different steps of the process might be considered

The activation of the HO-1/CO/H_2_S axis has the potential to improve the clinical course of Covid 19, including co-morbidities, and to prevent critical and fatal diseases. No treatment is currently validated for this purpose; however, several possible strategies are known. Treatments may target HO-1 expression, CO delivery, H_2_S endogenous synthesis, H_2_S delivery, and persulfide delivery, see Fig. 3.

#### Induction of HO-1

Increased expression of the *HMOX1* gene has been achieved by both natural substances and pharmacologic interventions. The best investigated natural substance that induces the enzyme is curcumin, a substance extracted from the rhizomes of the plant *Curcuma longa*. In experiments with H9c2 cells stressed with H_2_O_2_, curcumin at the concentration of 15 μM produced a 3-fold increase of HO-1 expression (Yang et al. 2017). However, the clinical use of curcumin is extremely questionable as it lacks the necessary solubility, selectivity and bioavailability and no clinical benefits from curcumin treatments have been recorded so far (Nelson et al. 2017).

Hemin (ferriprotoporphyrin IX), is a well-known inducer of HO-1 (Braggins et al. 1986) and is available as drug for human use, mainly for the treatment of acute intermittent porphyria. The administration of hemin at the dose of 20 mg/kg by intra-peritoneal injection to rats achieved significant increase of both HO-1 protein expression and function measured as increased production of bilirubin (Martın et al. 2019). More relevant, hemin at the dose of 30 mg/kg was able to increase HO-1 protein expression and to counteract acute lung injury induced by liver transplantation in rats (Chi et al. 2016). However, the therapeutic dose of hemin in porphyria is 3–4 mg/kg, i.e. far lower than the dose used in rats to achieve HO-1 induction, and toxicity is an issue already at the currently used doses (Anderson and Collins 2006). Moreover, the specific mechanism of toxicity likely in place with SARS-CoV-2, i.e. destruction of porphyrins (Liu and Li 2020v1; 2020v8), raises specific efficacy and safety issues with the use of hemin because the administered drug may provide further substrate for the viral aggression resulting in no efficacy and possible, unpredictable negative consequences.

#### Delivery of CO

Many investigational efforts are ongoing to validate treatments based on the delivery of pharmacologic amounts of CO. Several CO-Releasing Molecules (CORMs) and organic CO prodrugs have shown activity in experimental models. These substances are based on the ability of CO to reversibly bind transition metals carbonyl complexes and are made of such complexes linked to a pharmaceutically acceptable carrier that should drive the release at the place of need. They have shown activity in a variety of models of human pathology including lung injury (Ismailova et al. 2018). Unfortunately, CORMs carry significant safety issues due to their ability to generate carboxyhemoglobin, the risk of massive release of CO that is very toxic and the unknown destiny of the de-carbonylated CORM moiety. A new approach is the use of CORMs that are subject to some kind of endogenous activation so to actually release CO only at site of need, e.g. CORMs activated by increased local levels of reactive oxygen species or local acidosis (Ji and Wang 2018). However, none of the mentioned CORMs is approved for clinical use.

It is worth to note that some clinical findings in Covid 19 are vouching for a possible utility of CORMs. Indeed, cigarette smoking is an obvious way to introduce CO resulting in increased carboxyhemoglobin, which is among the negative health effects of smoking. In spite of an evident negative role of cigarette smoking in the clinical outcomes from any respiratory diseases, it has been consistently reported that the rate of smokers among Covid 19 patients is lower than expected based on the crude rate of smokers in the population and this discrepancy is confirmed also in severe clinical forms (Farsalinos et al. 2020; Rossato et al. 2020). A positive effect from the CO delivery from smoke is a possible explanation of these findings and would confirm that CO delivery is actually capable to modulate the aggressivity of Covid 19.

#### Support to endogenous synthesis of H_2_S

The carbon cycle and the transulfuration pathway are directly regulated by the availability of dietary micronutrients (Dattilo et al. 2016), thus it may be possible to boost the endogenous generation of H_2_S by administering selected micronutrients. Taurine supplementation has been shown to induce a significant reduction of blood pressure in parallel with an increased plasma concentration of H_2_S in pre-hypertensive subjects (Sun et al. 2016). In the same study taurine was also shown to increase the expression of the H_2_S producing enzymes, mainly CSE, in isolated human mesenteric arteries. Thereafter, being cysteine the main substrate for H_2_S synthesis, co-administration of taurine and N-acetylcysteine as cysteine donor was proposed as a better tool to promote cardiovascular health (DiNicolantonio et al. 2017). CSE activity is a lot more sensitive than CBS to the concentration of the co-factor pyridoxal-5-phosphate (vitamin B6) (Gregory et al. 2016) so that supra-physiological levels of vitamin B6 have the potential to further feed H_2_S release from CSE. We have been testing for precision medicine purposes a combination of micronutrients consisting of cysteines in the form of L-cystine, taurine and supraphysiologic amounts of vitamin B6 (pyridoxal 5-phosphate – P5P) and checked their effects on the metabolome by Liquid Chromatography Mass Spectrometry (LC-MS/MS) in several healthy subjects. These micronutrients consistently produced a metabolically relevant up-regulation of the products of H_2_S releasing reactions (data not shown) with a striking increase of the concentration of homolanthionine, which indicates a main activation of release of H_2_S from CSE, and a less evident but consistent increase of the other H_2_S metabolic markers, i.e. lanthionine (CBS activity) and sulfates (SO_4_^2−^, 3MST activity and H_2_S oxidation in mitochondria). This opportunity is attractive to investigate because micronutrients are widely available, easy and cheap to manufacture and potentially free from major side effects. However, it is uncertain whether such an intervention may be effective in inducing H_2_S within the lung and while a viral aggression is blocking the activity of HO-1 and the release of CO as argued based on Liu & Li (2020v1; 2020v8).

#### Delivery of H_2_S

The simplest way to deliver H_2_S is by inhalation but the necessary concentration would be toxic for the bronchial mucosa. Effective concentrations had been delivered to sheeps with partial cardiopulmonary bypass (Derwall et al. 2011), but this is unlikely to be a suitable clinical option.

In experimental models the delivery of H_2_S is usually achieved by the chemical donors, i.e. the sulfide salts sodium hydrosulfide (NaSH) and sodium sulfide (Na_2_S). These substances are very useful in tracking H_2_S effects in in-vitro systems and, to some extent, in animal models. However, they deliver massive amounts of H_2_S at once and are far away from replicating the necessary complex homeostatic regulation, which makes model-specific also the experimental data generated. For in vitro studies the problem may be addressed to some extent with microfluidic techniques (Christoforidis et al. 2018) but the clinical use of these substances remains unlikely.

Natural H_2_S donors occur in garlic and onions. Allicin, the most common of the thiosulfinates in garlic, decomposes into diallyl disulfide (DADS), diallyl sulfide (DAS), and diallyl trisulfide (DATS). All these substances have been shown to release H_2_S in vivo upon reaction with endogenous thiols on the membrane of human erythrocytes and to elicit a vasodilation response in parallel with H_2_S release in rats (Benavides et al. 2007). The clinical suitability of DATS, DADS, and DAS is limited due to their poor water solubility and to the generation of various by products (Powell et al. 2018). This has triggered pharmaceutical research and development of garlic derivates offering actual therapeutic opportunities, which is ongoing (Rose et al. 2019).

A variety of synthetic/pharmacologic H_2_S donors is under development, see Szabo and Papapetropoulos (2017) and Powell et al. (2018) for a review. A clinically suitable H_2_S donor should be water-soluble and stable under storage, should not generate toxic by-products and should have a specific and well-defined release mechanism possibly based on some kind of targeted activation of release. Compound GYY4137, which was used in the in vitro and in vivo models of antiviral activity of H_2_S (Ivanciuc et al. 2016; Li et al. 2015), is a hydrolysis triggered donor, i.e. it spontaneously releases H_2_S in aqueous solution although at a slower rate compared to NaHS. Other molecules under development include thiol-, light- and enzyme-triggered donors, but none of them is already available for clinical use.

#### Delivery of persulfides

It has been proposed that many of the actions of H_2_S, mainly persulfidation signalling, may be induced with the administration of biologically active persulfides, which has been done using sodium thiosulfate (Na_2_S_2_O_3_) (Bilska-Wilkosz et al. 2017). This is a stable, nontoxic metabolite of H_2_S that is clinically available for the treatment cyanide poisoning (injectable) and pityriasis versicolor (topical), limited to serious cases due to side effects. Sodium thiosulfate has been proposed as inhalation therapy for Covid 19 (Evgen’ev and Frenkel 2020), which sounds reasonable based on the above evidences, even though patients with advanced impairment of gaseous exchanges might not fully benefit from this route of administration.

It is however to be understood that any treatment able to upgrade the CO and/or H_2_S signalling may suffer a very narrow therapeutic window because excessive correction may easily result in toxic effects. The relationship between H_2_S and inflammation is controversial and seems to follow a biphasic regulation with either pro-inflammatory or anti-inflammatory effects depending on dose/method of activation, district of reference and target inflammatory cells so that it has been defined as a “double edged sword” (Hua et al. 2013). Some caution is necessary also looking at the role of H_2_S in infections. Besides the protective role against infections from capsulated RNA viruses, H_2_S was also shown to potentiate the resistance to antibiotics of several bacteria and to promote some invasion mechanisms of *Mycobacterium tubercolosis* (Pal et al. 2018) and these events might play a negative role in the clinical evolution of Covid 19.

### Summary and future directions

The defences of airways against enveloped RNA viruses include the activation of the HO-1/CO/H_2_S system and the release of H_2_S. A bulk of available evidences support the idea that the infection from SARS-CoV-2 responds to these mechanisms and the epidemiology, clinical manifestations, co-morbidities and risk factors for Covid 19 could be explained by a failure of this system. This understanding opens new avenues for the investigation of the disease, for the individuation of subjects at increased risk (e.g. *HMOX1* promoter polymorphisms) and for testing new treatments. A wide array of interventions with the potential to boost these natural defences is available or under clinical development. These include chemical, natural and pharmacological CO and H_2_S donors and also micronutrients have a potential to benefit. These opportunities should be prospectively investigated in the clinical setting. Meantime, the understanding of the above physiopathologic mechanisms may already be of guidance in the evaluation and tailoring of already implemented interventions with a special attention to the indication and modalities for mechanic ventilation and paracetamol administration.

## Data Availability

Not applicable.

## Abbreviations

H_2_S: Hydrogen sulfide
CO: Carbon monoxide
HO-1: Inducible Heme Oxigenase
*HMOX1*: Heme Oxigenase 1 gene
COVID-19: Coronavirus disease 2019
SARS-CoV-2: Severe acute respiratory syndrome coronavirus 2
NO: Nitric Oxide
RSV: Respiratory Sincitial Virus
HIV: Human Immunodeficiency Virus
CSE: Cystathionine Gamma Lyase
TAGDD: Thiol-Activated gem-Dithiol-based H_2_S donor
CBS: Cystathionine Beta Synthase
3MST: 3-Mercaptopyruvate Sulfur Transferase
GSH: Glutathione
LC-MS/MS: Liquid Chromatography Mass Spectrometry
ER: Endoplasmic Reticulum
DIC: Disseminated Intravascular Coagulation
MV: Mechanical Ventilation
VILI: Ventilation Associated Lung Injury
ACS: Acute Chest Syndrome
CORM: CO-Releasing Molecule
DADS: Diallyl disulfide
DAS: Diallyl sulfide
DATS: Diallyl trisulfide
